# Supplementary material for: Cloud BioLinux: pre-configured and on-demand bioinformatics computing for the genomics community
Source: BMC Bioinformatics. 2012 Mar 19;13:42. doi: 10.1186/1471-2105-13-42 (PMC3372431; doi:10.1186/1471-2105-13-42)
Supplement: Additional file 1 — Supplementary 1 Cloud BioLinux software documentation in the form of a mini, self-contained website. Users need to download and uncompress the .zip file, and open through a web browser the "index.html" file available on the main directory. (ZIP 1823 kb). [file 1471-2105-13-42-S1.ZIP › Cloud-BioLinux-Package-Documentation/docs/atv.html]

Bio-Linux Software Documentation Pages

Back to search form

## atv

|  |  |
| --- | --- |
| Name | atv |
| Description | **ATV (A Tree Viewer)** is a Java tool for the visualization of annotated phylogenetic trees. ATV reads standard New Hampshire format tree files (as produced by all major phylogenetic analysis software). ATV also implements an extended superset of New Hampshire format of our own design, called NHX format, which allows to extensively annotate a tree (a definition of NHX is available at: http://www.genetics.wustl.edu/eddy/forester/NHX.html).  **ATV** can be used both as an application and as an Applet. Two versions of ATV exist: One requires Java 2, the other one only requires Java 1.1.x (but has less functions). ATV is part of FORESTER (a framework for automated phylogenomics).  **References:**  Zmasek C. M. and Eddy S. R. (2001) ATV: display and manipulation of annotated phylogenetic trees. Bioinformatics, 17, 383-384. [PDF] |
| Homepage | http://www.genetics.wustl.edu/eddy/atv/ |
| Remote Documentation | http://www.genetics.wustl.edu/eddy/atv/atv\_documentation.pdf |
